# In Vitro and In Vivo Study of a Novel Porcine Collagen Membrane for Guided Bone Regeneration

**DOI:** 10.3390/ma9110949

**Published:** 2016-11-22

**Authors:** Eisner Salamanca, Chi-Yang Tsai, Yu-Hwa Pan, Yu-Te Lin, Haw-Ming Huang, Nai-Chia Teng, Che-Tong Lin, Sheng-Wei Feng, Wei-Jen Chang

**Affiliations:** 1 School of Dentistry, College of Oral Medicine, Taipei Medical University, Taipei 110, Taiwan; d204103004@tmu.edu.tw (E.S.); cytsai@tmu.edu.tw (C.-Y.T.); shalom.dc@msa.hinet.net (Y.-H.P.); dianaten@tmu.edu.tw (N.-C.T.); chetong@tmu.edu.tw (C.-T.L.); 2 Graduate Institute of Biomedical Materials & Tissue Engineering, College of Oral Medicine, Taipei Medical University, Taipei 110, Taiwan; hhm@tmu.edu.tw; 3 Dental Department, Taipei Medical University Hospital, Taipei 110, Taiwan; 4 Department of General Dentistry, Chang Gung Memorial Hospital, Taipei 106, Taiwan; 5 Graduate Institute of Dental and Craniofacial Science, Chang Gung University, Taoyuan 333, Taiwan; 6 Sunmax Biotech Co., Ltd., Tainan 741, Taiwan; yd@sunmaxbiotech.com; 7 Dental Department, Taipei Medical University, Shuang-Ho Hospital, Taipei 235, Taiwan

**Keywords:** collagen membrane, alveolar bone, guided bone regeneration, animal study

## Abstract

For years, in order to improve bone regeneration and prevent the need of a second stage surgery to remove non-resorbable membranes, biological absorbable membranes have gradually been developed and applied in guided tissue regeneration (GTR). The present study’s main objective was to achieve space maintenance and bone regeneration using a new freeze-dried developed porcine collagen membrane, and compare it with an already commercial collagen membrane, when both were used with a bovine xenograft in prepared alveolar ridge bone defects. Prior to surgery, the membrane’s vitality analysis showed statistically significant higher cell proliferation in the test membrane over the commercial one. In six beagle dogs, commercial bone xenograft was packed in lateral ridge bone defects prepared in the left and right side and then covered with test porcine collagen membrane or commercial collagen membrane. Alveolar height changes were measured. Histomorphometric results, in vitro and in vivo properties indicated that the new porcine collagen membrane is biocompatible, enhances bone xenograft osteoconduction, and reduces the alveolar ridge height reabsorption rate.

## 1. Introduction

Prior to the 1980s, patients with alveolar bone resorption due to periodontal or other oral diseases, without implementing bone regeneration treatment, exhibited minimal evidence of the recovery of hard and soft tissue to healthy levels. The human body is able to repair the majority of periodontal bony defects with long junctional epithelium, while other conditions are dependent on the regenerative ability of the bone, in which the healing process must be considered a repair mechanism, not regeneration [1]. During the initial stage of the healing process, there exists a competitive phenomenon among the cells from four different tissues: (1) gingiva tissue; (2) periodontal ligaments; (3) connective tissue; (4) cementum. Among them, the growth rate of 0.5 mm/day that gingival epithelial cells reach is substantially faster than the other three types of cells. Therefore, periodontal bony defects eventually heal as long junctional epithelium [2].

During the early 1980s, Nyman and Gottlow advocated the use of a non-absorbable expanded polytetrafluoroethylene (e-PTFE) membrane that was able to barricade the down-growth of gingival epithelial cells and create a space for new bone formation; Nyman and Gottlow named this technique guided tissue generation (GTR) [3,4]. The theoretical basis of the GTR technique was to obstruct the rapid growth of the gingival epithelial cells and simultaneously create a space for other cells to repopulate the bony defect area for further new bone formation. However, this particular barrier needed to be composed of a biologically compatible material. In 1986, Nyman and Gottlow first suggested the e-PTFE membrane as the barrier to be used for GTR, and the following clinical studies revealed the e-PTFE membrane to be stable and capable of maintaining space for bone regeneration [5,6]. In the following years, other types of membranes, such as the Teflon and Millipore membranes, were used in GTR, and new bone formation has been denoted both clinically and histologically [7,8,9].

With the purpose of regenerating bone, current therapies include multiple approaches such as onlay bone grafts, ridge splitting, regeneration, alveolar osteotomies/sandwich grafts, interpositional grafts, mandibular inferior border grafting, maxillary sinus floor elevation, growth factors, and use of subperiosteal membrane-guided regeneration. All of these procedures present advantages and disadvantages [10,11,12]. Non-absorbable membranes provide a good outcome; however, they present a pitfall for clinical purposes, which includes the necessity for removal during second-stage surgery. Another drawback, based on clinical and laboratory findings, was that the wounds tended to become inflamed and disrupt bone regeneration if there was an early exposure of the membrane during the healing process [13,14,15].

Recently, to prevent the need for second-stage surgery and possible infection due to membrane exposure, the biologically absorbable membrane was gradually developed and applied in GTR [16]. These absorbable membranes can be classified as collagen and non-collagen membranes [17]; these latter ones have, in some studies, demonstrated improved cell adhesion and achieved the growth rate using mineralized cellulose matrices [18]. In other studies, the non-collagen membrane sometimes lacked rigid support, and displayed bad clinical manageability. When the membrane has bovine origins, although no human cases of bovine spongiform encephalitis have been reported to date, some researchers are concerned about the long-term effects and the transmission of yet-unknown pathogenic proteins [19]. Collagen membranes are produced using processed animal collagen extracts from bovine, porcine, or other animals with 0.5 mm to 1 mm thickness for clinical use [20]. Clinical and histological findings have demonstrated an initial tissue regeneration after 5–7 days with a collagen membrane and complete absorption within a few weeks. Some studies during a one-year follow-up showed bone regeneration without evidence of immunological rejection [21,22,23].

Some advantageous properties of collagen over other materials include hemostatic function, allowing early wound stabilization; hemostatic properties to attract fibroblasts; and semipermeability that facilitates nutrient transfer. A major drawback of native collagen is the rapid biodegradation by the enzymatic activity of macrophages and polymorphonuclear leucocytes [24]. A novel process, using a freeze-dried technique, was used to develop the novel collagen membrane used in the present study to increase the membrane strength and prolong the resorption time. This freeze-dried technique was used to avoid damage to the collagen structure and any toxic residues. Therefore, the main goal of the present study was to achieve space maintenance and bone regeneration using a newly developed porcine collagen membrane when used with a bovine xenograft in prepared alveolar ridge bone defects.

## 2. Materials and Methods

### 2.1. Materials

The test membrane consisted of a monolayer porcine collagen membrane type I atelocollagen and the control membrane was a resorbable bilayer membrane, confirmed by highly purified non-cross-linked porcine type I and III collagen. They were stored at a room temperature, between 15 °C and 25 °C. The bovine xenograft was used at a particle size of 0.25 mm to 1 mm.

### 2.2. Scanning Electron Microscopy

A structural comparison of the layers and thickness, was done between the test membrane and the control one, using a scanning electron microscopy (SEM, HITACHI SU3500, Hitachi High-Technologies Corporations, Tokyo, Japan). The SEM was also used to evaluate the test and control membrane’s surface after being immersed for 3 days with MG-63 cells (3 × 10^3^ cells/well) in 500 µL Dulbecco’s Modified Eagle’s Medium.

### 2.3. MTT Assay

Cell metabolic activity was evaluated according to succinic dehydrogenase (SDH) activity using the spectrophotometric methyl tetrazolium assay (MTT assay). MG-63 cells were seeded (3 × 10^3^ cells/well) into 24-well plates (Costar Corp., Cambridge, MA, USA) with 500 µL Dulbecco’s Modified Eagle’s Medium and were maintained in a humidified incubator with 5% CO_2_ and 95% air at 37 °C for 24 h. Later, the culture medium was aspirated, and 500 µL of new media was added to all wells. In some wells, the 5 mm × 5 mm test membranes were inserted, and in other wells, control membranes of equal size were inserted. Control groups consisted of DMEM (Dulbecco’s Modified Eagle’s Medium) only and DMEM with 2% of Dimethyl sulfoxide (DMSO). After cultivation for 1, 3, and 5 days in a humidified incubator with 5% CO_2_ and 95% air at 37 °C, 50 µL of the MTT solution (5 mg/mL phosphate buffered saline—PBS) was added to all the wells and kept in the incubator for 4 h. Thereafter, all solutions were suctioned and replaced with 500 µL of DMSO. After 4 min, all solutions were transferred to a 96-well plate and the absorbance was measured at 570 nm with 690 nm as reference, using an ELISA reader (EZ Read 400, Biochrom, Ltd., Cambourne, UK).

### 2.4. Surgical Procedures

This study was approved by the Animal Care and Ethics Committee of Taipei Medical University. All experiments were performed in accordance with the guidelines laid down by the U.S. National Institutes of Health (NIH) regarding the care and use of animals for experimental procedures and the European Communities Council Directive of 24 November 1986 (86/609/EEC).

**Figure 1:**
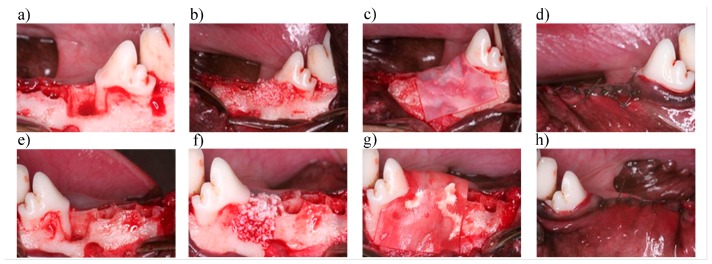
Surgical procedure for the treatment of bone defects in both groups. (**a**) Left bone box-shaped 5 mm × 5 mm defect prepared at the mesial side of the fourth premolar; (**b**) Xenograft placement within the defect; (**c**) The graft covered with the test collagen membrane; (**d**) primary closure was achieved. Steps in the surgical site; (**e**) Right bone box-shaped 5 mm × 5 mm defect prepared at the mesial side of the fourth premolar; (**f**) Xenograft placement within the defect; (**g**) The graft covered with the control membrane; (**h**) primary closure was achieved.

In this study, six beagles were used, exhibiting a mean age of 10.5 months ± 1.5 months and a weight of 8 kg ± 2 kg; there was an equal number of females and males. A split-mouth study design was used for the animals, and randomly, the left side was designed for the test membrane and the right side for the control membrane. General anesthesia consisted of Zoletil 50, and a dose of 10 mg/kg (Virbac Co., Carros, France) was administrated via intravenous injection. Routine dental infiltration anesthesia was used at each surgical site. After a local injection of 2% lidocaine (3M-ESPE, Neuss, Germany), the bilateral, mandibular second and third premolars were extracted, a mucoperiosteal flap was elevated, and a 5 mm × 5 mm defect was created with a round bur in a high-speed hand-piece and abundant irrigation. Bone defects were created at the mesial side of the first molar, and extended to the mid-buccal of the fourth premolar. Moreover, a concavity was created as a mark at the apical area of the defect. Then, the bovine xenograft material was first packed into the defect and the test collagen membrane was placed on top, covering 2 mm beyond the defect border; this procedure was repeated in all of the left bone defects. To treat all of the right bone defects, the bovine xenograft was packed, and the control membrane was placed on top; the control membrane also extended 2 mm beyond the defect border. Both of the surgical areas reached the primary closure with a simple, interrupted biodegradable suture (see in Figure 1). Each animal was held in a 1 m width × 1 m length × 1.2 m height cage in the animal center of Taipei Medical University at 21 °C ± 2 °C and 55% ± 20% humidity. After the surgery, a soft diet and water were supplied twice a day, 125 mg amoxicillin and 25 mg ketoprofen were given to the animals twice a day, and gentle brushing was performed once a day.

The beagles were sacrificed 4 and 8 weeks post-surgery. The bilateral fourth premolars accompanied by 5 mm of the surrounding bone were collected with a trephine bur. The collected samples were immersed in a 2.5% glutaraldehyde solution for 10 days and then moved into 4% EDTA solution for decalcification. The solution was renewed every 7 days and the samples were punctured with a needle after 2 weeks of decalcification. This process was considered complete if the samples could trespass. Once the samples were completely decalcified, they were stored in a 2.5% glutaraldehyde solution again and sent for sectioning. The completed histological specimens were then observed under a photomicroscope for histological and histomorphometric analysis.

## 3. Statistical Analysis

Pre-surgical and post-surgical changes in the defect height were measured with a periodontal probe, histological specimens were observed under a light microscope, histopathological changes were evaluated, and each histological slide was analyzed with Image-Pro Plus software to calculate the surface area ratio of the bone tissue, connective tissue, and residual bone grafts. A Student’s t-test was performed to compare the data from the MTT assay and an in vivo histomorphometric analysis. Results were considered statistically significant if *P* < 0.001 [25].

## 4. Results

**Figure 2:**
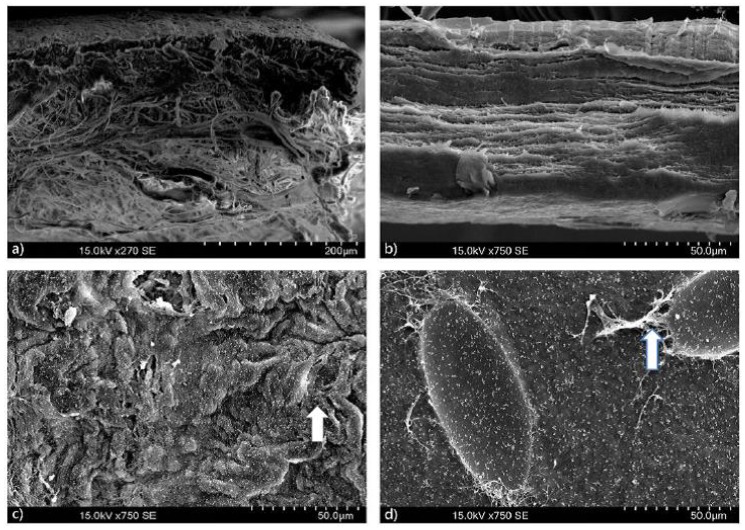
Scanning electron microscopy (SEM) showed: (**a**) Control membrane bilayer; (**b**) Test membrane monolayer; (**c**) Control membrane surface after 3 days with MG-63 cells in Dulbecco’s Modified Eagle’s Medium; (**d**) Test membrane surface after 3 days with MG-63 cells in Dulbecco’s Modified Eagle’s Medium.

In the scanning electron microscopy, it was possible to observe the higher thickness of the control membrane due to its double layer in comparison with the test membrane’s one layer. Three days after the membranes were cultured with MG-63 cells in Dulbecco’s Modified Eagle’s Medium, it was possible to see degradation of the surfaces and cell residues on top of the membranes (see in Figure 2).

**Figure 3:**
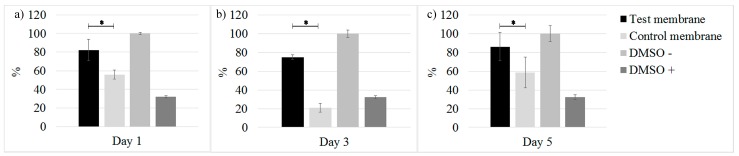
MTT assay. Vitality analysis comparison between both membranes at (**a**) 1; (**b**) 3 and (**c**) 5 days. * *P* < 0.001.

The MG-63 cell metabolic activity indicated that on day 1; the control membrane had a cytotoxic potential of 55.95% when compared to the 82.21% of the test membrane. On day 3, the test membrane exhibited 74.97% cytotoxic potential, which was better than the control membrane at 20.83%, a value even lower than the negative control. On day 5, the control membrane with 58.81% cytotoxic potential was improved over the negative control, but its performance remained lower than the 86.2% of the test membrane. The MTT assay results revealed a statistically significant difference *P* < 0.001, indicating that the test membrane exhibited a superior capacity over the control membrane to enhance cellular attachment, spreading, and viability (see in Figure 3).

Healing progressed uneventfully and there were no complications, such as allergic reactions, swellings, or any infections observed throughout the entire study period. All animals were sacrificed according to schedule.

**Figure 4:**
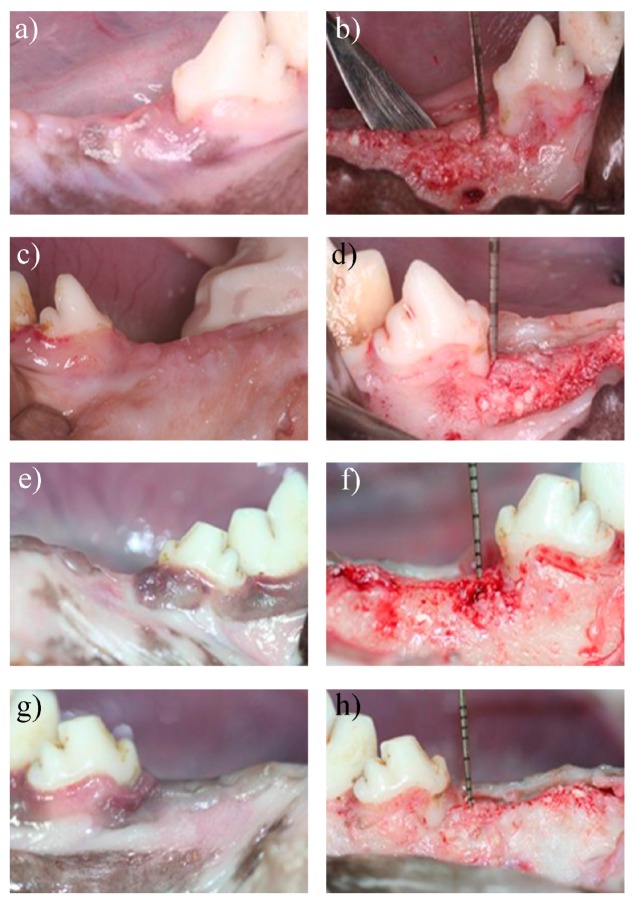
Surgical sites at 4 and 8 weeks post-surgery. In vivo observation of surgical sites and tissue regeneration at 4 and 8 weeks post-surgery. (**a**) Four weeks after surgery, the area treated with the test collagen membrane healed uneventfully; (**b**) Same surgical side from 4a image, the flap was elevated, and the bone defects demonstrated healing with a bone-like tissue; (**c**) Four weeks after the surgery, the area treated with the control membrane healed uneventfully; (**d**) The flap was elevated in same surgical side from 4c image, and the bone defect demonstrated healing with a bone-like tissue. Measurements from the cemento enamel junction (CEJ) showed the regained alveolar ridge height with a periodontal probe; (**e**) Eight weeks after surgery, the area treated with the test collagen membrane healed uneventfully; (**f**) Same surgical side from 4e image, the flap was elevated, and the bone defect demonstrated healing with a bone-like tissue; (**g**) Eight weeks after the surgery, the area treated with the control membrane healed uneventfully; (**h**) The flap was elevated in same surgical side from 4g image, and the bone defect demonstrated healing with a bone-like tissue. Measurements from the CEJ showed the regained alveolar ridge height with a periodontal probe.

After the first 4 weeks post-surgery, the wound healing process among the animals indicated that all tissues surrounding the surgery area were healthy. The primary closure reached with the sutures avoided collagen membrane exposure, and no inflammation was noted in the gingival coverage above the CEJ. Following the flap elevation, some graft particles were evident at the surgical site (see in Figure 4a–d).

During the eighth week of the healing process, some gingival recession was noted in both collagen membrane groups. At the time of reopening the surgical site, the tissue underneath appeared healthy, revealing an oral mucosa lined with a keratinized oral epithelium between the soft tissue and the bone. In addition, there was an increase in bone density, and some of the grafting materials had been replaced by mature regenerated bone (see in Figure 4e–h).

**Figure 5:**
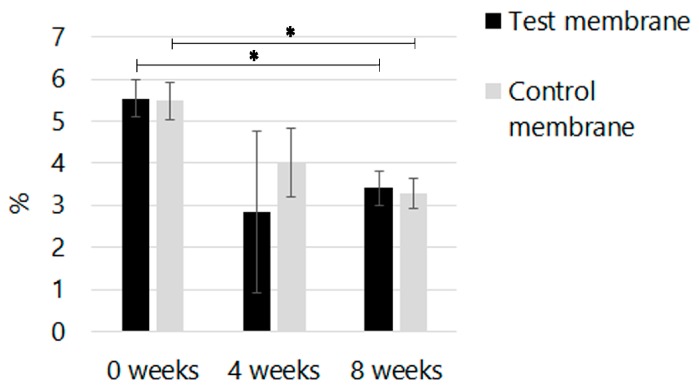
In vivo height changes of the alveolar ridge. Bone defects height changes at different time points, where 0 indicates surgery day. * *P* < 0.001.

Bone defects height changes: According to the data analysis and table of statistics, the bone defects height following GTR surgery in the present study did not indicate any significant differences between the test porcine collagen membrane and the control collagen membrane. This is likely due to the observation that both showed significant bone regeneration during the initial healing period and at 8 weeks, the regeneration remained almost similar. This result indicates that the placement of either the test porcine collagen membrane or the control membrane in GTR surgery associated with a bovine xenograft significantly helps to maintain the alveolar ridge height and promote new bone formation (see in Figure 5).

**Figure 6:**
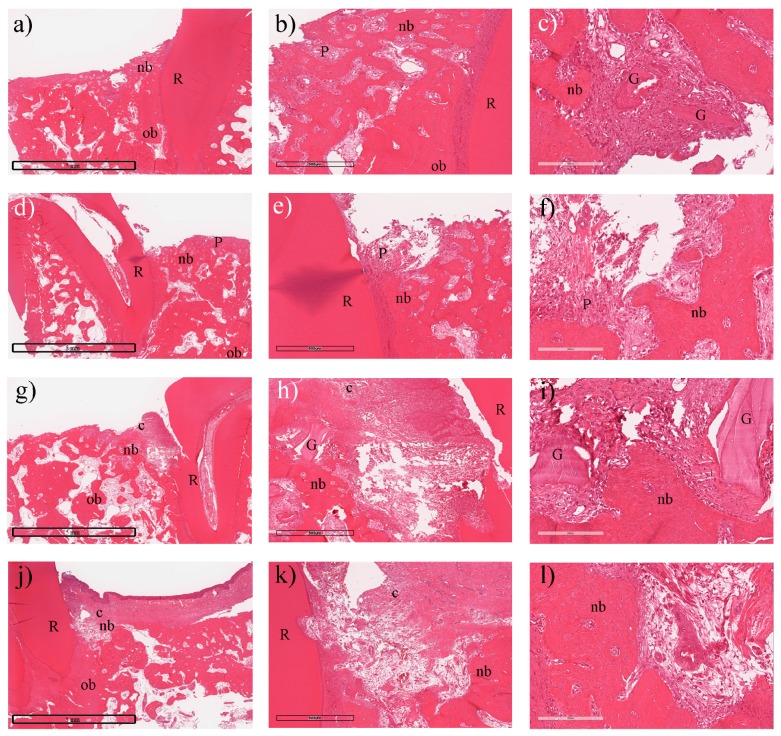
Histology and histomorphometric analysis (H&E stain): The use of both membranes showed similar behavior at 4 weeks post-surgery with residual collagen in the most coronal portion of the bone defect with woven new bone tissue and residual bone grafts filling the defect. Moreover, connective tissue surrounded some of the graft particles. (**a**–**c**) the test collagen membrane; (**d**–**f**) the control collagen membrane; In the test membrane group, (**a**) showed the intact test membrane (10×); (**b**) indicated new bone formation (nb) (80×) and (**c**) demonstrated residual bone grafts (G) (200×); In the control group, (**d**) showed the perforated membrane(10×); (**e**) performed new bone formation (nb); and (**f**) showed inflammation (P) without bone graft residual. At 8 weeks; (**g**) presented the test membrane (c) (10×); (**h**) showed the new bone formation (nb) without inflammation (80×) in the test group and (**i**) performed bone graft residual (G) (200×) under the test membrane; In the control group, (**j**) indicated the residual membrane (c) (10×); (**k**) showed the new bone formation (ob) surrounded with connective tissue (80×) and (**l**) also performed the higher magnification of (k) (200×).

According to the images of the histological specimen, the bone defects at 4 and 8 weeks after the GTR surgery, and the changes of the regained bone height were consistent with the clinical findings and were not affected by the type of collagen membrane that was used. Indeed, both membranes demonstrated a similar healing tendency, and there was little bone formation through the majority of the grafted site, with some woven bone and inflammatory tissues 4 weeks post-surgery (see in Figure 6a–f).

Eight weeks following surgery, the changes in bone height slowed and the bone graft particles started to become absorbed and were surrounded by new bone that extended from the walls and the apical border of the defect. During the same period, regeneration of the periodontal tissue and collagen membrane resorption occurred leaving some residual debris from the membranes. The bone density increased, and the bovine xenograft did not enhance bone formation but served as a scaffold for tissue formation during the healing process. Given the present results, it is difficult to judge whether the alveolar ridge height variations were complete or if an additional dimensional change may have occurred (see in Figure 6g–l).

Photographs of each histological specimen processed with the Image-Pro Plus software were taken to calculate the ratios for the surface area of bone formation, connective tissue, and residual bovine bone graft and to calculate their relative percentages. A comparison between test and control collagen membranes was also performed.

Four weeks post-surgery, the test and control membrane groups generated a similar percentage of new bone (27.7% ± 1.4%, and 27.3% ± 1.3%, respectively). The connective tissue was slightly higher in the membrane test group with 58.8% ± 6.6% compared to 55.2% ± 0.9% from the membrane control group. Residual particles from the bovine xenograft were lower in the membrane test group with 13.4% ± 5.2%, while the membrane control group exhibited 17.3% ± 2.3%. No significant statistical difference was found.

Eight weeks post-surgery, the test membrane had more new bone formation (34.0% ± 5.2%) over the membrane control group (30.7% ± 1.2%). The amount of connective tissue was slightly less in the membrane test group (53.0% ± 1.7%) than the membrane control group (55.7% ± 1.9%). The residual particles of the bovine xenograft were similar to the fourth week with less in the membrane test group at 12.9% ± 3.5%, while the membrane control group had 13.4% ± 3.2%. No significant statistical difference was found.

**Figure 7:**
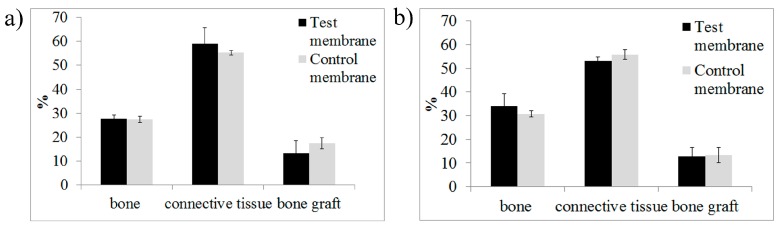
Percentage (%) distribution of the bone tissue, connective tissue, and residual bone grafts at (**a**) 4 and (**b**) 8 weeks.

Figure 7 shows the percentage distribution of the bone tissue, connective tissue, and residual bone grafts at four and eight weeks post-surgery with the use of the test collagen membrane and the control collagen membrane. At 4 weeks, a similar formation rate of bone tissue was noted in both groups. At 8 weeks, the test porcine collagen membrane group showed a superior bone formation rate than the group with the control collagen membrane. No significant statistical difference was found.

## 5. Discussion

Periodontal disease treatment refers to the removal of bacteria, the elimination of agents causing disease, and also implies the use of GTR to promote dentoalveolar tissue regeneration. The development and application of an absorbable collagen membrane in the experimental findings had also demonstrated proven efficacy for periodontal tissue regeneration [21,22,23]. Recently, animal and human studies have shown that GTR combining a xenograft with a collagen membrane for the treatment of periodontal bone defects could efficiently promote periodontal tissue repair and new bone formation [26,27,28]. One of the disadvantages of the currently developed collagen membranes is their unpredictable degree of resorption, which can significantly alter the amount of bone formation [17]. These drawbacks are time-consuming and risk the success of the GTR treatment. Therefore, novel collagen membranes had been developed to overcome these disadvantages and to promote a better GTR outcome. In the collagen membrane manufacturing process, heat compression and glutaraldehyde cross-linking are usually used to increase the strength and prolong the resorption time of the membrane. However, such heat compression could destroy the collagen structure and enhance toxicity due to residual glutaraldehydes. Therefore, a novel freeze-dried technique was used to develop the collagen membrane used in the present study to increase strength and prolong the resorption time. This freeze-dried technique does not damage the collagen structure or induce any toxic residues.

According to the results of this experiment, tooth extraction and mandibular bone atrophy was stimulated simultaneously by creating bone defects. After placing the xenograft material and covering it with both porcine collagen membranes, the healing condition of the soft tissue surrounding the defects at all observation times exhibited considerably favorable tissue integration and no inflammation was noted. In the present study, contamination and infection was avoided by a good primary closure which at the same time allowed good healing, graft material and membranes reduced reabsorption rate. These results are in concordance with Ronda et al. where they described a coronal advancement of the buccal flap in their study to reach a complete and stable closure of the flaps during any regenerative procedure [29].

Although there was a slight gingival recession in both test groups after 8 weeks, the histological analysis showed new bone formation and marked degradation of both collagen membranes during this period. This observation is in agreement with Shirakata et al. who also found that barrier membranes induce a complete integration of the bone grafts into new trabecular bone formed at the recipient site [30]. Similar results to those of the present study have also been found in humans studies where it is beneficial to use a combination of the commercial bone xenograft with the control membrane to limit the marginal ridge contraction following tooth extraction [31,32]. In the present study, our results indicate how similar, safe, and effective the test membrane is when combined with the bovine xenograft. In comparison with the control membrane, the test membrane also promoted osteoconduction when combined with the bovine xenograft; in another animal study, collagen membranes have enhanced bone regeneration, while space maintainance and membrane coverage were the two more important factors affecting graft bone regeneration [33]. The present study differs from that one by not having membrane exposure, which has shown to affect bone regeneration. All these results suggest the possibility of a similar performance from the test membrane to that of the control membrane and the commercial bone xenograft, an approach that has been analyzed multiple times in human studies for guided bone regeneration [34] or soft tissue support [35].

The control membrane used in previous human studies has been demonstrated to enhance guided bone regeneration and lateral ridge augmentation [36,37]. The test porcine collagen membrane used in the present study behaves in a similar manner to the control membrane; both were observed under the light microscope with the presence of inflammatory cells, indicating that the use of these absorbable membranes does not induce foreign body reaction and that they are biocompatible. Regarding clinical manageability, according to the only operator in the present study, both porcine collagen membranes were easy to manipulate, with a good wettability and hydrophilicity, allowing them to adhere and adjust to the area they covered. Both membranes are practical for clinical use, with the advantage of the test membrane over the control membrane being that there is no need to take care regarding which side faces the soft tissue.

This study differs from conventional animal experiment settings [26,38]. In particular, the tooth extraction and bone defects were prepared simultaneously in this study, despite some new tendencies of using recombinant human bone morphogenetic protein type 2 for more extensive reconstructions [39,40,41]. The present study focused on collagen membrane use, where the simultaneous extraction with bone defects preparation affected the wound healing process and caused more reduction of the newly formed bone at 4 weeks post-surgery. However, this phenomena ceased after 8 weeks when new bone formation and maturation was observed. It has been mentioned that different structural and physical characteristics, in conjunction with variable degradation times, may highly affect the regenerative outcome of membranes [20]. In the present study, both membranes presented similar regenerative outcomes, making the test porcine collagen membrane in this study a possible option for guided tissue regeneration for different periodontal treatments.

## 6. Conclusions

Within the limitations of this study, both the test membrane and the control collagen membrane demonstrate good biocompatibility and a similar bone formation rate when mixed with a commercial bone xenograft. Both were observed to enhance the osteoconduction properties of the graft material and reduce the alveolar ridge height reabsorption rate in bone defects. Moreover, the test membrane offers superior clinical manageability over the control collagen membrane. More extensive and controlled studies are needed to evaluate the test membrane.
